# Effect of Fibroblast Growth Factor-2 on Melanocyte Proliferation in Tissue-Engineered Skin Substitutes

**DOI:** 10.3390/ijms26041704

**Published:** 2025-02-17

**Authors:** Karel Ferland, Brice Magne, Henri De Koninck, Martin A. Barbier, Danielle Larouche, Lucie Germain

**Affiliations:** 1 The Tissue Engineering Laboratory (LOEX), Université Laval’s Research Center, Quebec, QC G1V 0A6, Canada; karel.ferland.1@ulaval.ca (K.F.); brice.magne@crchudequebec.ulaval.ca (B.M.); henri.de-koninck.1@ulaval.ca (H.D.K.); martin-alexandre.barbier.1@ulaval.ca (M.A.B.); danielle.larouche@crchudequebec.ulaval.ca (D.L.); 2 Department of Surgery, Faculty of Medicine, Université Laval, Quebec, QC G1V 0A6, Canada; 3 Regenerative Medicine Division, CHU de Québec-Université Laval Research Centre, Quebec, QC G1J 1Z4, Canada

**Keywords:** tissue engineering, melanocytes, skin, cell culture, tissue-engineered substitute

## Abstract

Burn patients treated with tissue-engineered skin substitutes (TESs) often experience pigmentation irregularities, including hypopigmentation and pigmentation spots. These issues are thought to stem from the reduced presence of melanocytes through dilution during TES manufacturing. To address this, we hypothesized that supplementing epithelial cell cultures—primarily composed of keratinocytes but also containing melanocytes—with Fibroblast Growth Factor-2 (FGF-2), a known promoter of melanocyte proliferation, could enhance melanocyte growth. This would potentially increase their numbers in TESs and improve pigmentation outcomes. Our findings indicate that FGF-2, at an optimal dose of 0.2 nM, effectively maintains melanocyte numbers in 2D cultures and epithelial cell cultures through the first passage. Importantly, this treatment does not interfere with keratinocyte proliferation or differentiation, nor does it affect TES integrity. However, FGF-2 supplementation alone did not increase the proportion of melanocytes in epithelial cultures beyond the first passage or in TESs. In summary, while FGF-2 supports melanocyte growth in culture, its addition alone was insufficient to significantly improve TES pigmentation.

## 1. Introduction

Tissue-engineered skin substitutes (TESs) are bilayered skin constructs consisting of fibroblasts and keratinocytes. These substitutes mimic the natural ability of cells to self-organize into a three-dimensional tissue structure without the need for external scaffolds or biomaterials. Thus, TESs closely resemble native human skin. TESs can be generated from autologous cells. Therefore, TESs are suitable for treating full-thickness skin injuries in patients with severe burns. The clinical TES product is named the Self-Assembled Skin Substitute (SASS). Clinical use of TESs has yielded promising results in burn patients, including 100% graft take, smooth and pliable skin, and the absence of hypertrophic scars [1]. However, the composition of TESs in terms of skin cell types may differ, with a reduced melanocyte-to-keratinocyte ratio compared to native skin. Moreover, structures present in native skin, such as hair follicles, glands, and nerves, are absent from TESs, leading to the loss of certain important functions.

Melanocytes are specialized cells predominantly located in the basal layer of the interfollicular skin epidermis and in the bulb region of the hair follicles [2]. These cells are responsible for skin pigmentation through melanin synthesis and transfer. Melanin synthesis, also known as melanogenesis, occurs within a specialized subset of lysosome-related organelles, the so-called melanosomes, that progressively mature into fully-melanized organelles [3]. Mature melanosomes are then transported to the tips of melanocyte dendrites and transferred to neighboring keratinocytes, where they accumulate as parasol-like structures on top of cell nuclei [4,5]. This process is believed to play a key role in preventing DNA damage in keratinocytes and reduce the risk of skin cancer development [6,7,8]. In healthy skin, the melanocyte-to-keratinocyte ratio is 1:40, and even distribution of melanocytes ensures uniform skin pigmentation [8].

**Figure 1:**
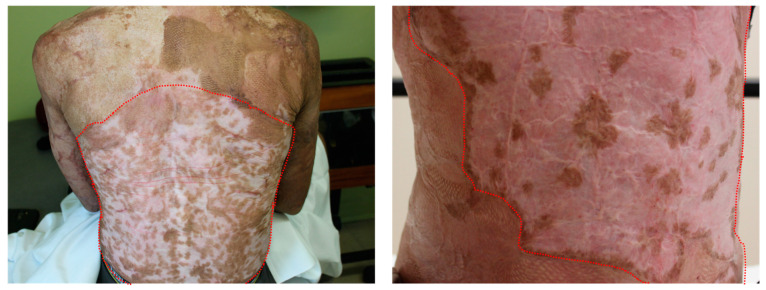
Pigmentation defects of patients grafted with TESs (SASSs). Back and abdomen of two patients grafted with SASSs. SASSs grafted areas are identified with red colored dots. Both patients present scattered pigmented spots across a large hypopigmented area after TES grafting.

In SASSs, however, minimal pigmentation is observed after transplantation, with only scattered spots appearing across grafted areas (Figure 1). While this patchy pigmentation indicates the presence of melanocytes in TESs, we hypothesized that their ratio to keratinocytes is heavily diminished throughout the TES production process, especially after serial sub-cultivation. This could occur because melanocytes are not purified during the production of TESs for burn patients but are still present in epithelial cell cultures. Keratinocytes are known to proliferate more rapidly than melanocytes; therefore, it is likely that melanocytes will be diluted over time in our current culture conditions [9]. Consequently, only a small number of melanocytes are present in TESs. They integrate into the epidermis and generate isolated pigmentation spots.

Pigmentation defects of bilayered skin substitutes after grafting are not limited to SASSs; other research teams also observed similar scattered spots and hypopigmentation at the sites grafted with skin substitutes [10,11,12]. To correct these defects, a few pre-clinical models of pigmented skin substitutes have been developed in recent years, all using the same approach: melanocytes are isolated from the epidermis, expanded separately from other cell types (mostly keratinocytes), and incorporated back into the skin substitutes [13,14,15,16,17,18]. These substitutes consistently exhibit uniform pigmentation after grafting on animals, suggesting that melanocytes in these pre-clinical models are functional as they produce melanin and transfer it to keratinocytes [13,14,15,16,17,18]. However, isolating and culturing melanocytes separately is a slow process that often involves the use of carcinogenic factors such as phorbol ester, making it impractical for clinical applications [19,20,21]. The majority of commercial media available on the market for melanocyte isolation and culture still use those carcinogenic factors, most commonly phorbol 12-myristate 13-acetate (PMA) [21]. While some PMA free media have been developed in the past few years and could potentially be used for clinical purposes [22], the process of isolating and the need to cultivate melanocytes separately remains tedious. Therefore, there is a need to develop a bilayered skin substitute, compatible with a clinical use, that contains enough melanocytes to improve pigmentation after grafting, thereby offering better treatment to burn patients. A more homogenous skin color could significantly contribute to their reinsertion into society and to enhance the quality of life of this most vulnerable population.

As an alternative to the approach described above (culturing keratinocytes and melanocytes separately), we hypothesized that melanocyte proliferation could be enhanced by supplementing the right factors to epithelial cell cultures as many keratinocyte isolation processes tend to retrieve other cell types present in the epithelium (epidermis and hair follicles), such as melanocytes. These factors should be naturally occurring molecules in the skin to physiologically stimulate proliferation while minimizing the risk of unwanted side effects during clinical applications. One such molecule is the Fibroblast Growth Factor-2 (FGF-2), a physiological growth factor secreted by fibroblasts in the skin, which promotes melanocyte proliferation via MAPK signaling [23,24]. FGF-2 can also stimulate keratinocyte growth, but its effect does not go beyond that of Epidermal Growth Factor (EGF) [25]. This makes FGF-2 an ideal candidate for selectively promoting melanocyte proliferation in epithelial cell cultures as keratinocyte medium used for amplification already contains EGF, which eclipses the effect of FGF-2 on keratinocytes [25]. Therefore, we evaluated the effects of FGF-2 on monolayer (2D) epithelial cell cultures and its potential to specifically enhance melanocyte proliferation. We also assessed its impact on TES structure and pigmentation when added during TES production in vitro. Increasing the melanocyte population in TES could lead to more uniform skin pigmentation for burn patients, resulting in improved aesthetic outcomes and better protection against UV radiation.

## 2. Results

### 2.1. FGF-2 Maintains Melanocyte Numbers in 2D Monocultures

**Figure 2:**
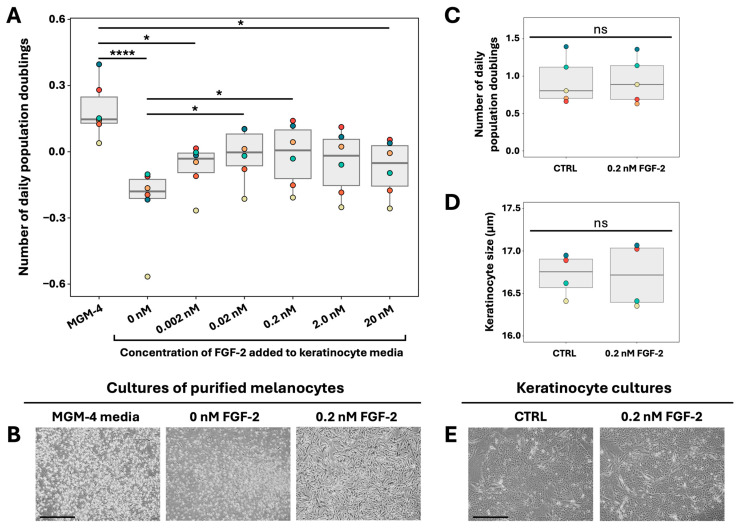
Effects of FGF-2 supplementation on melanocyte and keratinocyte 2D monocultures. (**A**) Daily population doubling of purified melanocytes cultured in 2D using keratinocyte medium supplemented with various doses of FGF-2. Melanocytes grown in commercial melanocyte medium MGM-4 were used as a positive control. (N = 6; * *p*-value < 0.05; **** *p*-value < 0.001). (**B**) Phase contrast microscopy of melanocytes cultured in different culture medium (40×, scale = 500 μm). (**C**) Daily population doubling of keratinocytes grown in 2D cultures using keratinocyte medium, either supplemented with the optimal FGF-2 dose (0.2 nM) or not (N = 5). (**D**) Average size of keratinocytes cultured with or without FGF-2 supplementation (N = 4). (**E**) Phase contrast microscopy of keratinocytes cultured with or without FGF-2 supplementation (40×, scale = 500 μm). Statistics: Boxplot represents the distribution of the mean value of each cell population. ns: not significant. Each colored dot represents the mean value of a cell population from one donor. The characteristics of different donors are presented in Supplementary Table S1. (**A**): Friedman test with Dunn’s multiple comparison; (**C**,**D**): *t*-test.

To initiate epithelial cell cultures (keratinocytes or melanocytes), the epithelium (epidermis and hair follicles) was separated from the dermis before dissociating the cells, resulting in the absence of fibroblasts in keratinocytes [26] and melanocytes cultures. To investigate the dose–response effect of FGF-2 supplementation on melanocyte proliferation, we cultured primary human skin melanocytes derived from human donors of diverse age groups, genders, anatomical sites and phototypes, as monoculture in keratinocyte medium containing varying concentrations of FGF-2, ranging from 0.002 nM to 20 nM. As a positive control, melanocytes were cultured in MGM-4, a commercially available melanocyte culture medium, for 7 days. Following the culture period, melanocytes were counted, and daily population doubling was calculated to assess proliferation. Melanocytes cultured in MGM-4 demonstrated significant growth after 7 days. In contrast, melanocytes cultured in keratinocyte medium with lower FGF-2 concentrations (<0.02 nM) showed negative daily population doubling, indicating that the mortality rate exceeded the proliferation rate (Figure 2A,B). However, the addition of FGF-2 to the keratinocyte medium at an optimal concentration of 0.2 nM significantly enhanced melanocyte population doublings, suggesting that it increased the proliferation rate sufficiently to offset cell death and therefore maintain melanocyte numbers in culture (Figure 2A,B). It was interesting to note that melanocytes isolated from light and dark skin phototypes did not respond differently to FGF-2; the blue and turquoise dots in Figure 2A represent dark-skin phototype melanocyte populations, while the others are light-skin phototype populations. Therefore, we did not compare light and dark skin phototypes for the following results. Melanocytes cultivated in keratinocyte medium express TYRP1, an enzyme expressed by melanocytes, 7 days after their seeding, confirming the identity of cells cultivated in keratinocyte medium as melanocytes (Supplementary Figure S1).

To ensure that FGF-2 does not affect keratinocyte proliferation and differentiation, we cultured primary human keratinocytes in keratinocyte medium containing 0.2 nM FGF-2 until they reach 95% confluency. We assessed daily population doubling using keratinocyte counts and evaluated the effect of FGF-2 on cell differentiation by measuring the mean cell size with a Beckman–Coulter cell counter as a larger cell size is associated with a more differentiated state in keratinocytes. At a dose of 0.2 nM, FGF-2 did not influence keratinocyte proliferation or differentiation as both cell proliferation and mean cell size remained unchanged following FGF-2 supplementation (Figure 2C–E).

### 2.2. FGF-2 Maintains Melanocyte Proportion in Epithelial Cell Cultures

**Figure 3:**
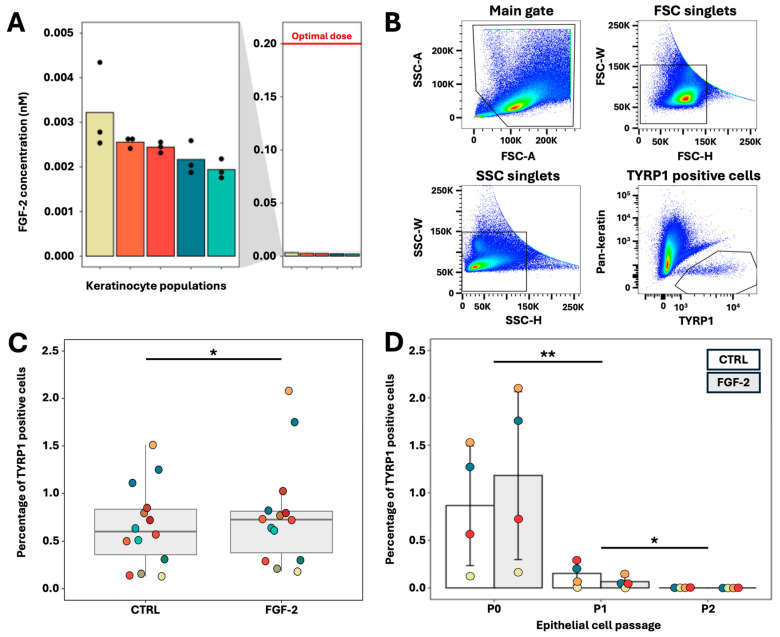
Effects of FGF-2 supplementation on epithelial cell cultures. (**A**) Quantification of FGF-2 secretion by keratinocytes (N = 5; n = 3) by ELISA. (**B**) Gating strategy for flow cytometry quantification of melanocytes (TYRP1+) in epithelial cell cultures. The bottom right graph shows the gate chosen to quantify the number of melanocytes (positive to TYRP1 and negative to pan-keratin). (**C**) TYRP1-positive cell quantification by flow cytometry in primary (P0) epithelial cell cultures. Note that the FGF-2 supplemented condition has a significantly higher percentage of melanocytes (TYRP1-positive cells negative for pan-keratin) than the control by paired *t*-test (N = 16; * *p*-value = 0.04956). (**D**) Melanocyte quantification of melanocytes by flow cytometry in primary (P0) epithelial cell culture or after 1 (P1) and 2 (P2) passages. There is a decrease in the percentage of melanocytes between P0 and P1, as well as between P1 and P2, by permutational ANOVA (N = 4; ** *p*-value = 0.009 (P0–P1) and 0.012 (P1–P2)). Statistics: Boxplot represents the distribution of the mean value of each cell population. (**A**) Bar plot represents mean values for technical replicate of each population. (**D**) Bar plots represent mean values with standard deviation (SD). Each colored dot represents the mean value of a cell population from one donor. The characteristics of different donors are presented in Supplementary Table S2. (**C**) *t*-test. (**D**) ANOVA by permutation with pairwise permutation *t* test multiple comparison.

Considering that FGF-2 promotes melanocyte but not keratinocyte proliferation, we hypothesized that culturing cells isolated from the epithelium in medium supplemented with FGF-2 would improve the melanocyte-to-keratinocyte ratio after serial sub-cultivation of keratinocytes. However, since keratinocytes are known to secrete FGF-2, we first measured the FGF-2 secretion by keratinocytes under our culture conditions (Figure 3A). This was accomplished using an ELISA assay on supernatants collected from keratinocytes cultured to 95% confluency. Keratinocytes secreted an average of 0.0022 nM FGF-2, a concentration that, according to our dose–response assay (Figure 2A), does not significantly enhance melanocyte proliferation. Consequently, for the remainder of our experiments, we used an optimal dose of 0.2 nM of FGF-2 as the secretion of FGF-2 by keratinocytes was considered negligible.

Next, to investigate the effects of FGF-2 on epithelial cell cultures, cells were isolated from the epithelium (epidermis and hair follicles) of human skin biopsies from various donors. Briefly, following its separation from the dermis using thermolysin, the epithelium was digested with trypsin-EDTA. The isolated cells were then seeded onto a feeder layer of irradiated human fibroblasts in keratinocyte medium supplemented with 0.2 nM FGF-2. After 5 days of culture, when the cells reached 70–90% confluency, they were trypsinized, and the percentage of melanocytes was determined by TYRP1 immunolabeling and flow cytometry quantification (Figure 3B and Supplementary Figure S2). A subset of these cells was reseeded for a second passage, after which the cells were trypsinized and flow cytometry was used to assess the melanocyte-to-keratinocyte ratio over serial sub-cultivation. We found that FGF-2 supplementation increased the melanocyte-to-keratinocyte ratio in cultures derived from freshly isolated cells (P0) (Figure 3C). However, in subsequent passages, there was no further increase in the melanocyte proportion despite culturing with continuous FGF-2 supplementation (Figure 3D). By the end of P2, melanocytes were nearly absent in the epithelial cell cultures, likely due to their slower proliferation rate compared to keratinocytes, leading to their progressive dilution over time.

### 2.3. FGF-2 Treatment Does Not Affect TES Integrity

**Figure 4:**
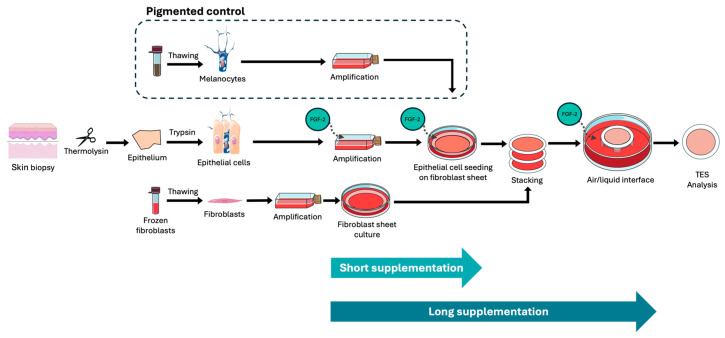
TES production protocol. The epithelium (epidermis and hair follicles) is recovered from skin biopsy, and epithelial cells are isolated and cultured in the presence of FGF-2. Epithelial cells are seeded onto fibroblast sheet from previously thawed fibroblast. One epithelial sheet is stacked onto other fibroblast sheets and cultured at the air–liquid interface before analysis. To generate pigmented control, exogenously amplified melanocytes are also added to the TES.

**Figure 5:**
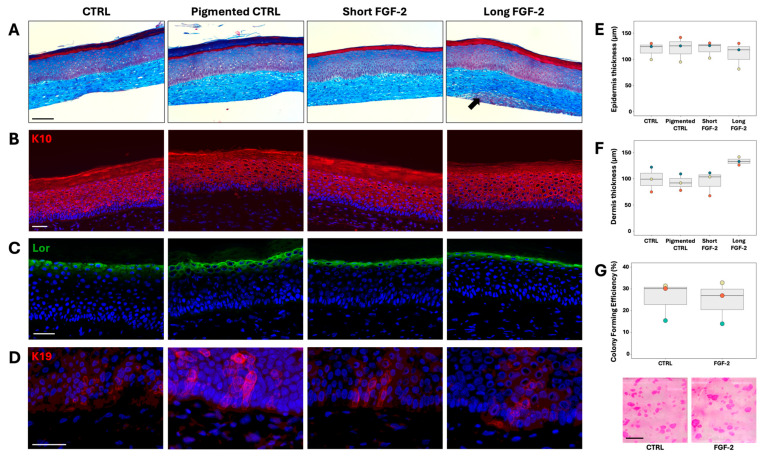
Effects of FGF-2 supplementation on TESs. (**A**) Masson’s trichrome staining of TESs after 10 days of culture at the air–liquid interface. The arrow indicates a group of fibroblasts in the dermis dermal portion in contact with the medium (100×; scale = 100 μm). Immunofluorescence of TESs against (**B**) keratin (K) 10 (red), (**C**) loricrin (Lor) (green), and (**D**) keratin 19 (red). Cell nuclei were stained with Hoechst (blue) (200×; scale = 50 μm). (**E**) TES epidermis average thickness (N = 3). (**F**) TES dermis average thickness (N = 3). (**G**) Colony forming efficiency of epithelial cells used to produce TES epidermis and macroscopic colony appearance (N = 3; rhodamine staining; scale = 1 cm). Statistics: Boxplot represents the distribution of the mean value of a cell population from one donor. The characteristics of different donors are presented in Supplementary Table S3. (**E**,**F**) Friedman test. (**G**) *t*-test.

To investigate the potential of FGF-2 to increase melanocyte numbers in TES, we generated TESs using freshly isolated human epithelial cells cultured either with FGF-2 (FGF-2 group) or without it (control group). These cells were seeded at passage 1 onto tissue-engineered dermis prepared from neonatal fibroblasts. During the culture at the air–liquid interface, FGF-2 was added only for the FGF-2 long supplementation group. For comparison, TESs were also cultured without FGF-2 supplementation at the air–liquid interface (short supplementation group). Additionally, pigmented TESs were produced by seeding purified melanocytes previously amplified in monoculture separately before adding keratinocytes (Figure 4). Our observations indicated that FGF-2 supplementation did not influence the formation of the epidermis (Figure 5A). Markers of keratinocyte differentiation, such as Keratin 10 (suprabasal layers) and loricrin (granular layer), were similarly expressed in both short-term and long-term FGF-2-treated TESs at levels comparable to the controls (Figure 5B,C). The epidermal thickness was not affected by FGF-2 treatment (Figure 5E). Additionally, FGF-2 did not alter the stem cell population in epithelial cell cultures, as evidenced by the comparable number of colonies in FGF-2-treated and control cultures (Figure 5G).

Both short- and long-term FGF-2-treated TESs also contain cells expressing keratin 19, a stem cell marker in the epidermis (Figure 5D). However, long-term FGF-2 supplementation appears to increase dermal thickness (Figure 5F), likely due to enhanced fibroblast proliferation. A great number of fibroblasts were observed at the base of the TES in long-term FGF-2-treated TESs (Figure 5A arrow).

### 2.4. FGF-2 Treatment Does Not Increase Melanocyte Numbers in TESs

**Figure 6:**
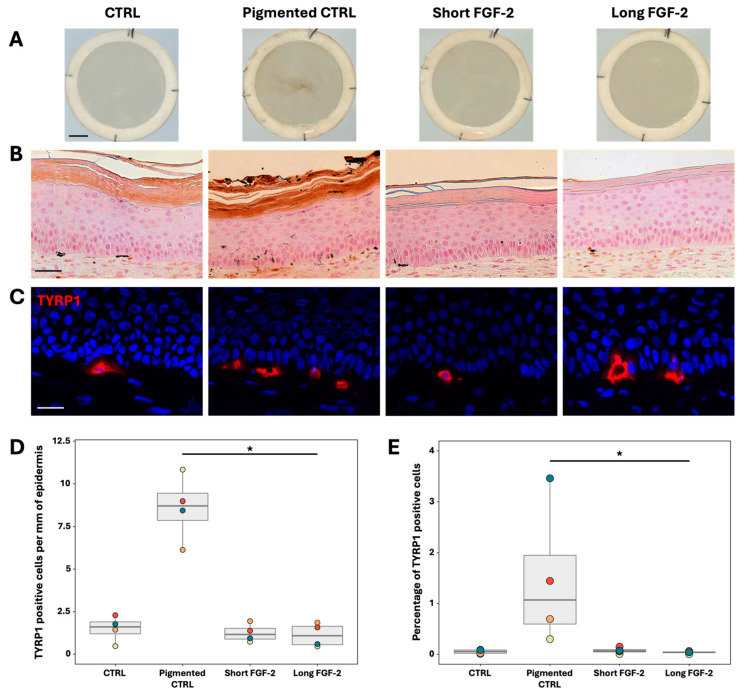
Effects of FGF-2 supplementation on the melanocyte concentration in TESs. (**A**) Macroscopic appearance of TESs after 10 days of culture at the air–liquid interface (scale = 1 cm). (**B**) Warthin–Starry staining of melanocytes in TESs after 10 days of culture at the air–liquid interface (200×; scale = 50 μm). (**C**) TYRP1 (red) immunofluorescence labeling of TESs. Cell nuclei were stained with Hoechst (blue) (400×; scale = 50 μm). (**D**) Quantification of melanocytes in TESs using TYRP1 immunofluorescence labeling of tissue sections. Note that the number of melanocytes in the pigmented control is significantly higher than in TESs cultured with long FGF-2 supplementation (N = 4; * *p*-value = 0.037). (**E**) Quantification of TYRP1 positive cells in the epidermis of TESs by flow cytometry (N = 4). The pigmented control count is significantly higher than TESs cultured with long term FGF-2 supplementation (N = 4; * *p*-value = 0.0155). Statistics: Boxplot represents the distribution of the mean value of a cell population from one donor. The characteristics of different donors are presented in Supplementary Table S4. (**D**,**E**): Friedman test with Dunn multiple comparison.

Pigmentation and melanocyte presence were evaluated in TESs produced as described in Figure 4. Macroscopic observations reveal that TESs treated with FGF-2 do not exhibit pigmentation compared to the pigmented control (Figure 6A). Nevertheless, melanocytes were detected by immunofluorescence in all TESs conditions (Figure 6B). These melanocytes were primarily located at the very base of the epidermis, with some appearing to even be on the dermal side. However, a preliminary analysis showed that melanocytes are indeed within the epidermis as they are positioned above the basement membrane of the TES. Quantification of both immunofluorescence and flow cytometry data revealed that FGF-2 treatment does not increase the number of melanocytes per millimeter or their quantity in TESs compared to the controls (Figure 6C,D).

## 3. Discussion

In this study, we assess the effect of Fibroblast Growth Factor-2 (FGF-2), a physiological factor, as a potential supplement to increase the number of melanocytes in Tissue-Engineered Skin substitutes (TESs). We show that FGF-2 maintains melanocytes cell populations in 2D purified melanocyte monocultures and in 2D epithelial cell cultures up to the end of the primary culture (P0). However, this effect diminishes with prolonged culture. By the end of the first (P1) and second passages (P2), melanocytes are scarce in epithelial cell cultures. Furthermore, FGF-2 stimulation shows no impact on TESs pigmentation, as the melanocyte proportion is comparable between treated and untreated conditions.

FGF-2 was selected due to its ability to promote melanocyte proliferation [23,24,27]. Additionally, preliminary experiments comparing various physiological factors, chosen for their potential influence on melanocytes, identified FGF-2 as the most promising candidate amongst other tested factors, which included stem cell factor (SCF), granulocyte-macrophage colony-stimulating factor (GM-CSF), and endothelin-1 (ET-1) (Supplementary Figure S3) [28,29,30,31]. Incidentally, FGF-2 is already used in clinics for vitiligo treatment [32]. Indeed, patients with vitiligo express a lower level of FGF-2 mRNA. Topical application of FGF-2 helps to re-pigment the hypopigmented areas by stimulating melanocyte migration.

FGF-2 maintains melanocytes in 2D cultures at an optimal concentration of 0.2 nM. While the ability of FGF-2 to stimulate melanocyte proliferation has been previously reported [23,24], it is interesting to note that melanocytes can be maintained in our keratinocyte medium with the addition of FGF-2 alone. In this environment, melanocytes exhibit a spindle-shape phenotype, contrasting with melanocytes cultured in MGM-4, who exhibit a more dendritic phenotype. This spindle shape could indicate that these melanocytes secrete less melanin since this phenotype is associated with less differentiated melanocytes [33,34,35]. This may imply that something is lacking in the keratinocyte medium to induce melanin synthesis and dendricity during 2D culture. Nonetheless, these melanocytes are still differentiated enough to express TYRP1, an enzyme present in late-stage melanosome. Moreover, they still secrete melanin, as can be seen in TESs, where melanocytes are colored with Warthin–Starry staining (Figure 6B), a coloration which marks melanin with silver salts [36].

We employed flow cytometry to determine the percentage of melanocytes in epithelial cell cultures, with TYRP1 as a specific marker for melanocytes. The proportion (percentage) of melanocytes among other epithelial cells (mostly keratinocytes) was used to study the effect of FGF-2 on melanocyte growth, as it is an easy value for comparison among different conditions. However, it is difficult to find a reference value for the percentage of melanocytes in the epidermis because their number varies significantly depending on anatomical sites, age, and gender [37]. In children, the range goes from 3 to 12% of epithelial cells, while for adults, the range is between 4 and 26% of epithelial cells which are melanocytes. In our cultures, at the end of P0, we had up to 2% of melanocytes, with the highest proportions of melanocytes being in the FGF-2 treated condition. However, the effect of FGF-2 was not sufficient beyond the first passage as the proportion of melanocytes at the end of P1 and P2 is very low. This can likely be attributed to the high proliferation rate of keratinocytes, which significantly outpaces melanocyte proliferation by an important margin, resulting in the dilution of melanocytes in epithelial cell cultures. If so, this would explain why the positive effect of FGF-2 observed on melanocyte growth (Figure 2A and Figure 3C) is not maintained. It would also imply that the gap in proliferation rates between keratinocytes and melanocytes cannot be bridged by the addition of FGF-2 alone. Incidentally, the calculated percentages of melanocytes for P1 and P2 (Figure 3D) may fall within a margin of error, as they were so low, which might explain why the percentage of melanocytes in epithelial cells treated with FGF-2 at the end of the first passage (P1) appears lower than the control (Figure 3D).

FGF-2 treatment does not increase melanocyte numbers in TESs, despite its promising effects in primary 2D cell cultures. We hypothesized that here again, under our culture conditions, keratinocytes proliferate too rapidly, and the increase in melanocyte proliferation is insufficient to offset the significantly faster growth rate of keratinocytes. It is well established that melanocytes have a slow growth rate and exhibit limited proliferation under physiological conditions, unlike keratinocytes, which must continuously renew to maintain the epidermal cell turnover [9,38]. In fact, all pigmented skin substitutes developed in recent years rely on the addition of exogenously amplified melanocytes [13,14,15,16,17,18]. These substitutes are most used in a pre-clinical setting for which the addition of carcinogenic factors, such as phorbol esters, to induce melanocyte proliferation is not a limitation. While it is possible to culture melanocytes without phorbol esters [39] (some commercially available media already exist), in this study, we demonstrated that melanocytes can be cultured in the medium developed for keratinocytes without the need to isolate them from other epithelial cells simply by adding FGF-2. However, the effect of FGF-2 alone seems insufficient to increase the melanocyte proportion to a percentage that can improve TES pigmentation. It is important to note that we did not quantify pigmentation in TESs, other than macroscopically, in this study. It has been demonstrated that pigmentation improves in TESs after grafting onto athymic mice [13] or on human (Figure 1). Thus, it is likely that pigmentation could have improved after grafting the TESs. In the future, it would be interesting to analyze TES pigmentation after grafting onto athymic mice, as well as to use other physiological factors and combine them with FGF-2 to manufacture a culture medium that is optimal for both keratinocytes and melanocytes. This approach could significantly streamline the production process for clinical applications as it would eliminate the need for separate cultures, which can be costly and time consuming.

Ultimately, our study showed the capacity of FGF-2 to increase melanocyte proliferation in short-term 2D epithelial cell culture and hinted at its potential to enhance melanocyte proportion in TESs if combined with other factors to reduce the dilution effect by keratinocytes. FGF-2 also proved to have no impact on keratinocyte proliferation and differentiation, nor to affect TESs formation, which makes it an ideal additive without side effects. Further experiments will be required to test out new factor combinations in order to advance melanocyte culture and explore new avenues of improvement for TESs. Another interesting option could be to slow down keratinocyte proliferation to match melanocyte growth in culture, therefore minimizing dilution. A fully pigmented TESs could enhance burn patient treatment by offering better protection against UV radiation and a more homogeneous skin color.

## 4. Materials and Methods

### 4.1. Ethical Considerations

These experiments were conducted in accordance with the Declaration of Helsinki and the guidelines of our institution. The study received approval from our institution’s ethic committee for human participants (Comité d’éthique de la recherche du CHU de Québec—Université Laval: No. 2012-1248 and No. 2012-1251 initially approved on 28 April 2012 and renewed annually since). Written informed consent was obtained for the use of excised skin tissues for research purposes.

### 4.2. Cell Isolation and Culture

Human cells were isolated from healthy skin samples collected from various anatomical sites and excised during elective surgeries (Supplementary Tables S1–S4). Keratinocytes, melanocytes, and fibroblasts were isolated as previously described [13] from specimens with Fitzpatrick types estimated between II and V. Briefly, skin biopsies were washed 10 times in sterile phosphate buffered saline (PBS) (Thermo Fisher Scientific, Ottawa, ON, Canada) supplemented with antibiotics (100 U/mL penicillin (Sigma-Aldrich, St. Louis, MO, USA) and 25 µg/mL gentamicin (Gemini Bio, West Sacramento, CA, USA)) and cut into thin (1 to 3 mm wide) strips. Then, biopsies were incubated in 500 µg/mL thermolysin (Sigma, Oakville, ON, Canada) solubilized in HEPES buffer (0.01 M 4-(2-hydroxyethyl)-1-piperazineethanesulfonic acid (HEPES; MP Biomedicals, Santa Ana, CA, USA solutions with 0.67 mM KCl (Thermo Fisher Scientific), 0.14 M NaCl (Thermo Fisher Scientific) and 1 mM CaCl_2_ (Sigma Aldrich, Saint-Louis, MO, USA)) and incubated at 4 °C for 16 h to enzymatically digest the basement membrane. The epithelium (epidermis and hair follicles) was then peeled from the dermis using pliers. Epithelial cells were dissociated in a trypsin (0.05% *w*/*v*) (Gibco, Grand Island, NY, USA)-EDTA (0.01% *w*/*v*) (Sigma) solution for 25 min at 37 °C under constant agitation using a magnetic stirrer (DWK Life Sciences, Wertheim, Germany). The resulting suspension was passed through a 70 µm cell strainer (Corning, New York, NY, USA) to remove large tissue debris and centrifuged (300× *g*, 10 min). The resulting cell pellet was resuspended to generate the epithelial cell suspension and split into different culture flasks. To obtain pure melanocyte cultures, epithelial cells were seeded in culture flasks containing Melanocyte Growth Medium-4 (MGM-4) (Lonza, Kingston, ON, Canada), designed to enhance melanocyte proliferation. Melanocyte culture medium was supplemented with geneticin at 100 µg/mL (Sigma Aldrich), which is toxic to keratinocytes but not melanocytes as geneticin is harmful for cells with high division rates like keratinocytes [40]. The combination of melanocyte culture medium and geneticin specifically selects melanocytes, allowing for the generation of a pure melanocyte culture from the epithelial cell suspension. In parallel, the epithelial cell suspension was also seeded on a feeder layer of irradiated human fibroblasts maintained in keratinocyte medium (Dulbecco-Vogt modified Eagle medium (Gibco™, Waltham, MA, USA): Ham’s F12 (Gibco™), ratio 3:1, supplemented with 24.25 μg/mL adenine (Sigma-Aldrich), 5 μg/mL insulin (Sigma-Aldrich), 0.4 μg/mL hydrocortisone (Galenova, Saint-Hyacinthe, QC, Canada), 0.212 μg/mL isoproterenol hydrochloride (Sigma-Aldrich), 5% bovine HyClone FetalClone II serum (GE Healthcare, Chicago, IL, USA), 10 ng/mL human epidermal growth factor (Austral Biologicals, San Ramon, CA, USA), and antibiotics. Lastly, the dermis was digested in 0.125 U/mL of collagenase H (Roche Diagnostics, Laval, QC, Canada) for 3 h at 37 °C under agitation. Debris were removed using a 70 µm cell strainer and cells were pelleted through centrifugation. The dermal cells were seeded and cultured in fibroblast medium (Dulbecco–Vogt-modified Eagle medium (Gibco™, Waltham, MA, USA) supplemented with 10% Avantor Seradigm FB Essence serum (AvantorR, Radnor, PA, USA) and antibiotics).

### 4.3. TES Production

TESs were generated as previously described [41,42]. Briefly, fibroblasts were seeded at a density of 8 × 10^3^ cells/cm^2^ onto a 28.27 cm^2^ culture-treated petri dish (Corning). A hollowed anchoring paper (grade 610 filter paper, Ahlstrom, Taylorville, IL, USA) was placed at the base of the dish to facilitate handling of the reconstructed tissue. Fibroblasts were cultured for 21 days in fibroblast medium supplemented with 50 μg/mL of ascorbic acid (Sigma). Subsequently, keratinocytes were seeded at a density of 100,000 cells/cm^2^ onto the resulting dermal sheets. The dermal sheets with keratinocytes were then cultured in keratinocyte medium containing 50 μg/mL of ascorbic acid for 4 days before stacking the sheet onto two additional dermal sheets to enhance dermal thickness. TESs were cultured at the air–liquid interface for 10 days in EGF-free keratinocyte medium supplemented with 50 μg/mL of ascorbic acid.

### 4.4. Immunofluorescence

Tissues were embedded in OCT compound (Sakura Finetek, Torrance, CA, USA), flash-frozen in liquid nitrogen and stored at −80 °C. Sections (5 µm thick) were cut using a cryostat (Leica, Wetzlar, Germany) and fixed in pure acetone for 10 min at −20 °C. Tissue sections were blocked for 30 min in 2% bovine serum albumin (BSA) solubilized in calcium- and magnesium-enriched phosphate-buffered saline (IFPBS). Between each incubation step, tissue sections were washed for 2 min, 3 times in IFPBS. Tissue sections were incubated for one hour in the dark, with antibodies resuspended at their working concentration (Supplementary Table S5) in IFPBS supplemented with 2% BSA. Immunolabelled tissue sections were observed using a confocal (LSM700, Zeiss, Toronto, ON, Canada) or immunofluorescence microscope (Axio Imager M2, Zeiss).

### 4.5. Flow Cytometry

Cells were detached from culture flask using trypsin (0.05% *w*/*v*)-EDTA (0.01% *w*/*v*) or from skin substitutes using the method described above for cell isolation. Cells were resuspended and fixed in the fixation buffer for 20 min, permeabilized in permeabilization buffer (Intracellular Fixation and Permeabilization Buffer Set; eBioscience, Waltham, MA, USA) and incubated with antibodies diluted at their working concentration in PBS with 0.5 mM EDTA + 2% *v*/*v* human albumin (Sigma) + 5 µg/mL human IgG (Sigma) (Supplementary Table S5). Immunolabelled cells were analyzed using the Melody Flow Cytometer (BD Bioscience, Franklin Lakes, NJ, USA). At least 10,000 cells were analyzed. Debris and cell doublets were excluded from the analysis based on forward- and side-scattering signals. Gates were placed based on the immunofluorescence signal of appropriate negative controls (cells stained with isotype negative control antibody). Data was analyzed using FlowJo (v.10.7.0, LLC, BD).

### 4.6. FGF-2 ELISA Quantification

Keratinocytes were seeded at 6500 cells/cm^2^ in keratinocyte culture medium. When keratinocytes reached 45–50% confluency, the culture medium was changed to a new keratinocyte culture medium and conditioned for two days. FGF-2 was quantified from conditioned culture medium using a commercially available ELISA (human FGF basic ELISA kit; R&D, Minneapolis, MN, USA).

### 4.7. Histology

TES biopsies were formalin fixed, and paraffin embedded. Five µm sections were cut with a microtome (Leica), stained with Masson’s trichrome staining or Warthin–Starry staining and analyzed with a Axio Imager M2 (Zeiss). Epidermis and dermis thickness were measured using ImageJ Software (v1. 53j) [43].

### 4.8. Statistical Analysis

Friedman’s tests, followed by Dunn’s multiple comparison tests, were performed using GraphPad Prism version 8.0.0 for Windows (GraphPad Software, Boston, MA, USA, www.graphpad.com). Paired *t*-test and ANOVA were performed using statistical software R (v3.6.1, R Studio v1.1.456) and the package RVAideMemoire (v0.9-83-2). Figures were generated using the package ggplot2 (v3.5.1) [44].

## Data Availability

The data presented in this study are available upon request to the corresponding author.

## Supplementary Materials

The following supporting information can be downloaded at https://www.mdpi.com/article/10.3390/ijms26041704/s1.
