# Supplementary material for: Effect of Fibroblast Growth Factor-2 on Melanocyte Proliferation in Tissue-Engineered Skin Substitutes
Source: Int J Mol Sci. 2025 Feb 17;26(4):1704. doi: 10.3390/ijms26041704 (PMC11855325; doi:10.3390/ijms26041704)
Supplement: Supplementary file 1 [file ijms-26-01704-s001.zip › ijms-3429630-supplementary.pdf]

Supplementary Table S1: Cell populations used in figure 2

Cell Type	Identifier	Source	Sex	Age	Phototype	Color in graphs
Melanocyte	MelAabF2146X	Abdomen	Female	46 y.o.	Light	Yellow
	MelBsaaF1653X	Arm	Female	53 y.o.	Light	Light orange
	MelMaaH1369X	Breast	Female	69 y.o.	Light	Orange
	MelPEsE130.1Y	Foreskin	Male	1 month	Light	Red
	MelPEvC200.1Y	Foreskin	Male	1 month	Dark	Blue
	MelPEzE130.1Y	Foreskin	Male	1 month	Dark	Turquoise
Keratinocyte	KArJ2050X	Abdomen	Female	50 y.o.	Light	Yellow
	KMaaH1369X	Breast	Female	69 y.o.	Light	Orange
	KPEsE130.1Y	Foreskin	Male	1 month	Light	Red
	KPEvC200.1Y	Foreskin	Male	1 month	Dark	Blue
	KPEzE130.1Y	Foreskin	Male	1 month	Dark	Turquoise

Supplementary Table S2: Cell populations used in figure 3

Cell Type	Identifier	Source	Sex	Age	Phototype	Color in graphs
Keratinocyte	KAadK2239Y	Abdomen	Male	39 y.o.	Light	Yellow
	KAuD2238X	Abdomen	Female	38 y.o.	Light	Light orange
	KMuD2238X	Breast	Female	38 y.o.	Light	Orange
	KPEsE130.1Y	Foreskin	Male	1 month	Light	Red
	KPEzE130.1Y	Foreskin	Male	1 month	Dark	Blue
Epidermal cell	EpiPEdG230.1Y10JF3	Foreskin	Male	1 month	Dark	Orange
	EpiPEdG230.1Y7JF4	Foreskin	Male	1 month	Dark	Red
	EpiPEkG230.2YF2	Foreskin	Male	2 months	Light	Blue
	EpiPElC240.1YF2	Foreskin	Male	1 month	Light	Light blue
	EpiPElC240.2YF5	Foreskin	Male	2 months	Dark	Turquoise
	EpiPElC2427JF4	Foreskin	Male	1 month	Dark	Olive
	EpiPEmB230.1Y3JF4	Foreskin	Male	1 month	Dark	Brown
	EpiPEmB230.1YF3	Foreskin	Male	1 month	Dark	Dark Orange
	EpiPEmB230.2Y8JF2	Foreskin	Male	2 months	Light	Dark Red
	EpiPExA230.2Y1JF3	Foreskin	Male	2 months	Dark	Light orange
	EpiPExA230.2Y1JF4	Foreskin	Male	2 months	Dark	Red
	EpiPExA230.2Y3JF5	Foreskin	Male	2 months	Dark	Blue
	EpiAadK2239Y	Abdomen	Male	39 y.o.	Light	Yellow
	EpiFtB2359XF2	Buttock	Female	59 y.o.	Light	Dark turquoise

Supplementary Table S3: Cell populations used in figure 5

Cell Type	Identifier	Source	Sex	Age	Phototype	Color in graphs
Epidermal cell	EpiMadI2143X	Breast	Female	43 y.o.	Light	Yellow
	EpiAuD2238X	Abdomen	Female	38 y.o.	Light	Orange
	EpiPEkG230.2YF2	Foreskin	Male	2 months	Dark	Blue
	EpiMvD2144X	Breast	Female	44 y.o.	Light	Turquoise

Supplementary Table S4: Cell populations used in figure 6

Cell Type	Identifier	Source	Sex	Age	Phototype	Color in graphs
Epidermal cell	EpiFtB2359XF2	Buttock	Female	59 y.o.	Light	Yellow
	EpiPEdG230.1Y10JF3	Foreskin	Male	1 month	Dark	Light orange
	EpiPEdG230.1Y7JF4	Foreskin	Male	1 month	Dark	Red
	EpiPEkG230.2YF2	Foreskin	Male	2 months	Light	Blue

Supplementary Table S5: List of antibodies used

Use	Target	Clone	Specie	Conjugate	Supplier	Catalog Number	Dilution
Immuno-fluorescence	TYRP1	Ta99	Mouse	-	Novus Biologicals	NBP2-34720	1/1000
	Keratin 10	Polyclonal	Rabbit	-	Milipore Sigma	HPA012014	1/200
	Keratin 19	A53-B/A2	Mouse	Cy3	Gift of U. Karsten, Institute of biological sciences, University of Rostock (Germany)	-	1/200
	Loricrin	Poly19051	Rabbit	-	BioLegend	905104	1/1000
	Rabbit	Polyclonal	Goat	AF594	Invitrogen	A11012	1/1600
	Mouse	Polyclonal	Goat	AF594	Invitrogen	A11005	1/1400
	Rabbit	Polyclonal	Donkey	AF488	Invitrogen	A21206	1/1600
Flow cytometry	TYRP1	Ta99	Mouse	AF488	Novus Biologicals	NBP2-34720AF488	1/300
	Pan-Keratin	C-11	Mouse	PE	abcam	ab52460	1/100

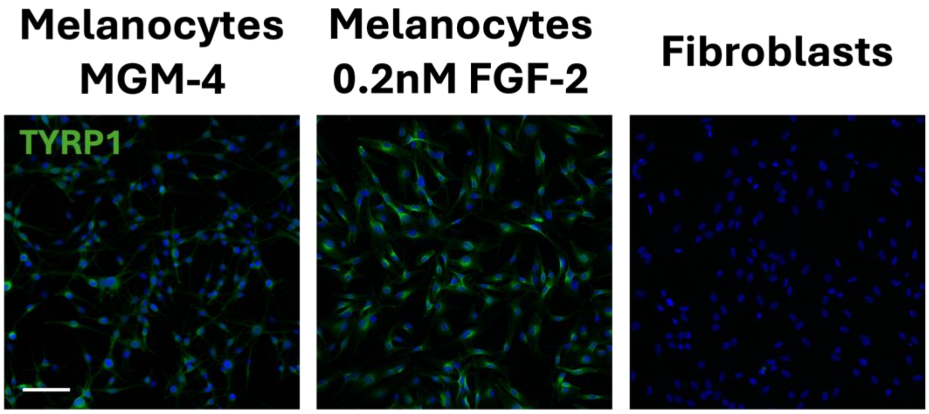

Supplementary Figure S1: TYRP1 expression of melanocytes and fibroblasts. Immunofluorescence of melanocytes cultivated in MGM-4 media and melanocytes and fibroblasts cultivated in keratinocytes media supplemented with 0.2nM FGF-2 (100X, scale =100 μm) (N=1).

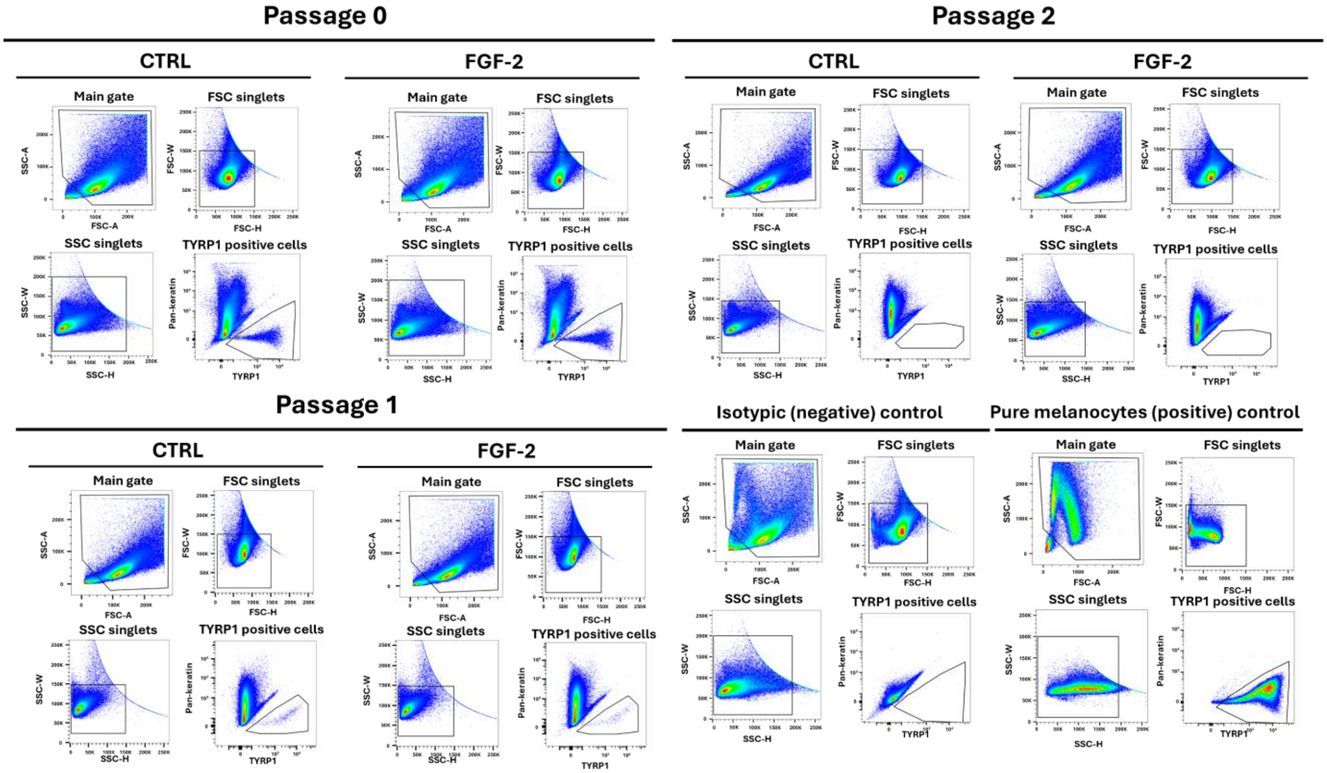

Supplementary Figure S2: Gating strategies of epithelial cells and controls. Gating strategies of flow cytometry graphs of control and FGF-2 treated epithelial cells at passage 0, 1 and 2 with isotypic (negative) control and pure melanocytes (positive) control of passage 0 epithelial cells (N=1).

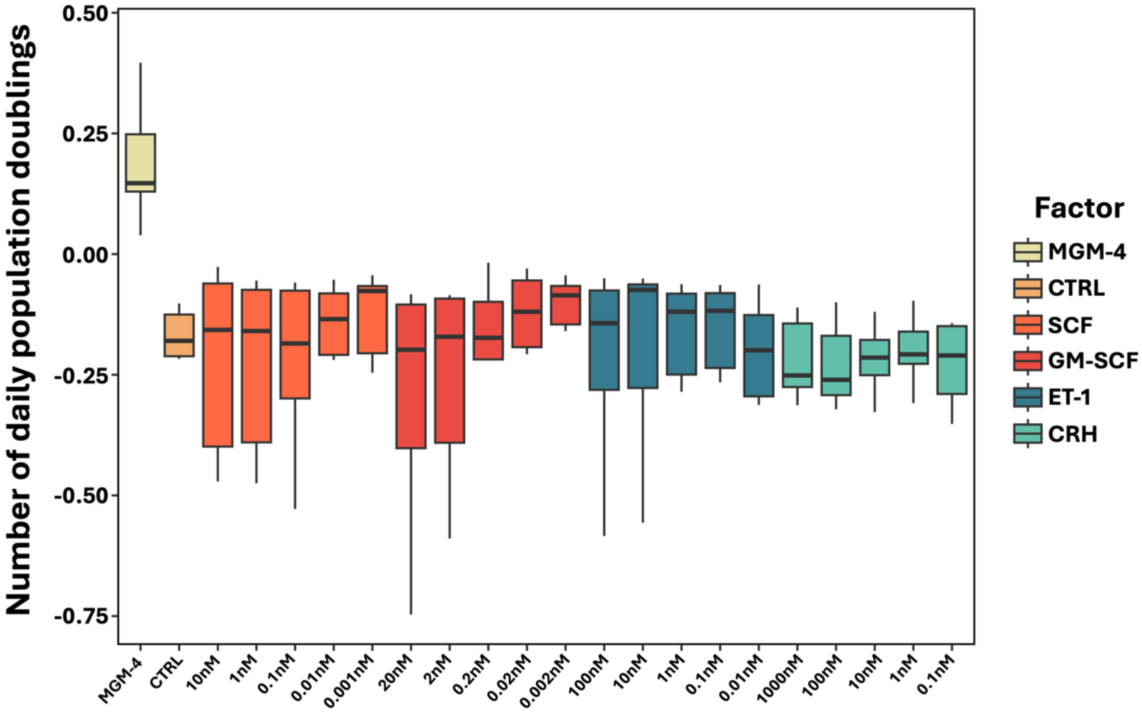

Supplementary Figure S3: Effect of physiological melanogenic factor in keratinocyte media on number of daily population doublings of melanocytes. Number of daily population doublings of melanocytes cultured in keratinocyte media with stem cell factor (SCF), Growth Macropphage-Colony Stimulating Factor (GM-CSF), Endothelin-1 (ET-1) and Corticotropin Relasing Hormone (CRH) (N=5 à 6).
